# Hydrolysis of three different head groups phospholipids by chicken group V phospholipase A2 using the monomolecular film technique

**DOI:** 10.1042/BSR20192053

**Published:** 2020-01-21

**Authors:** Aida Karray, Madiha Bou Ali, Jallouli Raida, Bezzine Sofiane

**Affiliations:** Laboratoire de Biochimie et de Génie Enzymatique des Lipases, Ecole Nationale d’Ingénieurs de Sfax, route de Soukra, Sfax 3038, Université de Sfax, Tunisia

**Keywords:** binding surface, lipid monolayer, penetration capacity, Phospholipase A2 group V

## Abstract

The kinetic aspects of lipolysis by pulmonary phospholipase A2 (ChPLA2-V), chicken intestinal phospholipase A2 (ChPLA2-IIA) and chicken pancreatic phospholipase A2 (ChPLA2-IB), from chicken have been compared using the monomolecular films technique, on short-chain phospholipids (with three different head groups) and on long-chain phospholipids.

The main conclusions from our experimental data indicate that the maximum catalytic activities of ChPLA2-V on 1,2 phosphatidylcholine and 1,2 phosphatidylethanolamine reached 15.26 and 36.12 moles/cm^2^.min.mM, respectively, at a pressure of 15 and 35 dynes/cm, respectively. Whereas, those of ChPLA2-IB were 3.58 (at the pressure of 20 dynes/cm) and 4.9 moles/cm^2^.min.mM. However, hydrolysis of phosphatidylglycerol monolayers (C12PG), were very much higher compared with all the substrates tested with 122 moles/cm^2^.min. Surprisingly, the hydrolysis rate of ChPLA2-V on long-chain phosphatidylglycerol (C18PG) was very low (1.45 moles/cm^2^.min) compared with all tested substrates, even with the use of *p*-cyclodextrin. And thus, the fatty acid preference of ChPLA2-V was 2-decanoyl > 2-oleoyl with a PG head group.

In order to gain significant correlations between enzyme’s structures and their relative functions, we tried to examine the surface electrostatic potentials of the various secreted phospholipase 2 (sPLA2) from chicken. In the present study, we detailed that the substrate affinity, specificity and the hydrolysis rates of sPLA2 at each interface is governed by the surface electrostatic potentials and hydrophobic interactions operative at this surface.

## Introduction

Phospholipases A2 (PLA2) are lipolytic enzymes that act on phospholipids at the sn-2 position generating free fatty acids and lyso-phospholipids [1]. With respect to their structural features, cell distributions and their functions, the PLA_2_ classes differ strongly from each other. Subgroup of secreted PLA2s (sPLA2s) includes pancreatic group IB (sPLA2-IB), non- pancreatic group II (subgroups A–F), group III, V, X and XII (subgroupsA–B) [2].

Clearly, the different mammalian sPLA2s are not isoforms as their sequence identities are only approximately 15% [3,4], they have distinct enzymatic properties [5] and show different tissue distribution patterns in both mice and humans [6]. Consequently, in various tissues, the different sPLA2s may exert distinct biological functions that may be dependent or independent of their enzymatic activities [3,7,8].

sPLA2 catalyses the hydrolysis of sn-glycero-3-phospholipids at sn-2 position, thus producing 1-lysophospholipid and a free fatty acid, e.g., arachidonic acid, which takes part in cell signalling. Nevertheless, several mechanisms of physiological reactions diverge from bulk (emulsified system) to bidimensional states (monolayer study). In fact, the monolayer methodology has been used to compare the relative surface activities of proteins or its interactions with lipid monolayers spread over an aqueous subphase. Much less is known about the regulation and biological roles of phospholipases A2 from birds. A recent study by Karray et al. focussed their study on the identification of novel phospholipases A2 from chicken using biochemical and molecular techniques [23]. These studies include: purification and characterisation of two active enzymes from chicken, interfacial catalytic properties characterisation of chicken pancreatic sPLA2 (ChPLA2-IB) and chicken intestinal sPLA2 (ChPLA2-IIA), acting on three different phospholipids spread as monomolecular films, isolation of new genes encoding several sPLA2, evaluation of the relative expression level of these genes in tissues and/or organs extracted from healthy chicken then chicken with lung inflammation: avian infection bronchitis.

The main result of the present study indicates that for chickens with infectious bronchitis, an overexpression of pulmonary PLA2 (ChPLA2-V) was observed in lungs and spleen in comparison with healthy chickens. These findings suggest that ChPLA2-V could be a potential biomarker for lung inflammation.

Then, group V sPLA2 has been cloned from chicken human, rat and mouse species [9,10]. Structure features of this sPLA2 show that contrary to group I and II sPLA2s, group V sPLA2 has only six disulfides, defining a novel group of sPLA2s [11]. The highest identity level of this sPLA2 was obtained with group IIA sPLA2s, as compared with several sPLA2 groups. It was also devoid of the N-propeptide sequence, characterising group I and group X sPLA2, strongly approving the close relationship with group II sPLA2s.

In the present work, we characterised for the first time, the interfacial catalytic properties of ChPLA2-V, acting on three different phospholipids heads group spread as monomolecular films. To examine the substrate preference of the ChPLA2-V, variations with surface pressure of the catalytic activities of chicken group VsPLA2 were tested using zwitterionic I-2 didodecanoyl-sn-glycero-3-phosphatidylcholine (1,2 DDPC), partially negative charged 1,2 didodecanoyl-sn-3-phosphatidy-ethanolamine (1,2 DDPE) and negatively charged 1,2 PG. We aimed also to test the capacity of chicken sPLA2 group V to act on short-chain fatty acids on the phospholipid backbone, in order to gain more structural–functional relationship of ChPLA2-V. For further comparison, ChPLA2-IB and ChPLA2-IIA were also tested under the same experimental conditions.

## Materials and methods

### Chemicals

NaCl, CaCl_2_, Tris/HCl, Ethylene Diamine Tetra Acetic acid (EDTA), β-cyclodextrin (β-CD) were purchased from Sigma–Aldrich (St. Quentin-Fallavier, France). Chloroform supplied from SDS (Peypin, France) was used as the spreading solvent.

### Phospholipids

1,2 DDPC, 1 palmitoyl-2-oleoyl-sn-3- phosphatidyl-glycerol (1,2 POPG) and β-CD were purchased from Sigma–Aldrich (St. Quentin-Fallavier, France). 1,2 DDPE, 1,2 didodecanoyl-sn-3-phosphatidy-glycerol (1,2 DDPG) was from Avanti Polar Lipids. All substrates are used without further purification, and their surface compression isotherms were performed as described below.

### PLA2

ChPLA2-IB and ChPLA2-IIA were purified from pancreatic juice and intestinal mucosa, respectively, as previously described [12,13].

The fully active recombinant ChPLA2-V was expressed in *Pichia pastoris* and purified. Whereas, pure ChPLA2-IB and IIA were native enzymes.

All the enzymes (ChPLA2-IB, IIA and V) were used at a concentration of 1 mg/ml.

### Pressure–area curves

To obtain information on the behaviour of phospholipids tested, surface pressure–molecular area curves were drawn up for zwitter-ionic 1,2 DDPC, partially negatively charged 1,2 DDPE and negatively charged 1,2 PG at different surface pressures. Typically, 25 μl (1 mg/ml) of phospholipid in a chloroformic solution were spread on to a ‘zero-order’ Teflon trough filled with 200 ml of 150 mM NaCl,10 mM Tris, pH 8, 21 mM CaCl_2_, 1 mM EDTA. The film was relaxed and subsequently compressed to the target pressure. The collapse and the other phase-transition points were estimated by the third derivate method [14]. The results of these experiments are plotted in a graph of surface pressure versus mean area per molecule.

### Monomolecular film technique for kinetic measurements on sPLA2

The monolayer study was performed as described previously by Pattus et al. [15]. Prior to each experiment, the Teflon trough used to form the monomolecular film was cleaned with water before being gently brushed in the presence of distilled ethanol and washed again with tap water. The aqueous subphase contained 10 mM Tris/HCl, pH 8, 150 mM NaCl, 21 mM CaCl_2_, and 1 mM EDTA with all phospholipases tested. The buffer was prepared with double-distilled water and filtered through a 0.22-μm Millipore filter. Kinetic experiments were performed at room temperature with a KSV-2200 barostat (KSV Helsinki) and a ‘zero-order’ Teflon trough equipped with a mobile Teflon barrier, which was used to compensate for the substrate molecules removed from the film by enzyme hydrolysis, thus maintaining the surface pressure constant. The latter was measured using a Wilhelmy plate (perimeter 3.94 cm) attached to an electro-balance, which was connected in turn to a microprocessor controlling the movements of the mobile barrier. The subphase of the reaction compartment was continuously agitated with a 2-cm magnetic stirrer moving at 250 rpm to assure a homogeneous distribution of the enzyme during the reaction. Before each experiment, the Teflon trough used for forming the monomolecular film is cleaned with water, then gently brushed in the presence of distilled ethanol, washed again with tap water, and finally rinsed with double-distilled water. Then, we filled the trough with a buffer solution. Any residual surface active impurities were removed before each assay by sweeping and suctioning the surface. Finally, we spread the substrate (phosphlipids solution) on the surface of both compartments. The enzyme solution (5–100 μl) was injected through the film over the stirrer with a Hamilton syringe. The surface area of the reaction compartment was 108.58 cm^2^ and its volume was 130 ml. The reservoir compartment was 148 mm wide and 249 mm long. After injection of the enzyme in the reaction compartment, the enzyme kinetics were recorded during 10–20 min, at room temperature, then a second Teflon barrier was placed in-between the two compartments to stop the film flow from the right to the left surface.

By catalysing phospholipid using monomolecular film technique, sPLA2 released 1- lysophospholipid and a free fatty acid. These hydrolytic products were much more soluble than the phospholipid substrate, spread on monolayer film. Thus, they desorb on the aqueous phase, leading to a surface pressure decrease. Consequently, enzymatic activity was characterised as surface pressure changed at constant total area. Enzymatic units were expressed and defined as the number of moles of the appropriate substrate hydrolysed by unit time and unit surface (mol.cm^−2^.min^−1^) of the reaction compartment of the ‘zero-order’ trough for an arbitrary sPLA2 concentration.

## Results and discussion

### Variations with surface pressure of the catalytic activities of chicken group V; IB and IIA sPLA2 using DDPC, DDPE, DDPG and POPG as substrate

The maximum catalytic activities of ChPLA2-V on monomolecular film technique were studied with three different phospholipid head groups spread in the form of monomolecular films at the air–water interface. To examine the substrate preference of the ChPLA2-V, three different head groups (PC, PE, and PG) were tested. Both long chains fatty acid and short chains on the phospholipid backbone were also compared (2-oleoyl and 2-octanoyl phosphatidyl-glycerol) with ChPLA2-V.

For the sake of comparison, enzymes from chicken: ChPLA2-IB, and ChPLA2-IIA were also used under the same experimental conditions. In order to solubilise water-insoluble long chain lipolytic products (C18:1), 0.5 mM of β-CD, a lipolytic products acceptor, was added to the aqueous subphase, when 1,2 POPG was used as substrate.

The data obtained showed a clear capacity of ChPLA2-V to hydrolyse phosphatidyl-choline (a zwitterionic head group substrate), phosphatidyl-ethanolamine and phosphatidyl-glycerol (a partially negatively and negatively charged substrate, respectively). Whereas, phosphatidyl- glycerol was found to be the best substrate tested. But when we tested the ChPLA2-V activity on long-chain phosphatidyl-glycerol (1 palmitoyl-2-oleoyl-sn-3-phosphatidyl-glycerol) the hydrolysis rate was very low compared with all the substrates tested (Table 1).

**Table 1:** The different enzymatic activities (moles/cm^2^.min.mM) of each PLA2 enzyme and PL substrates at the corresponding maximum pressure (dynes/cm) and molecular area

sPLA2 group	Substrate	Enzymatic activity (moles/cm^2^.min.mM)	Max pressure (dynes/cm)	Molecular area (moles/cm^2^)
**V**	DDPC	15.26	15	183764E-10
	DDPE	36.12	35	320783E-10
	POPG	1.45	35	184382E-10
	DDPG	**122**	25	**16504E-10**
**IB**	DDPC	3.58	20	198581E-10
	DDPE	4.9	20	269297E-10
	POPG	**14.3**	35	**184382E-10**
	DDPG	3.6	30	17644E-10
**IIA**	DDPC	1.11	15	183764E-10
	DDPE	6.158	25	285138E-10
	POPG	**10.5**	35	**184382E-10**
	DDPG	12	25	16504E-10

Bold P-values correspond to the maximum enzymatic activity and the corresponding molecular area.

**Figure 1:**
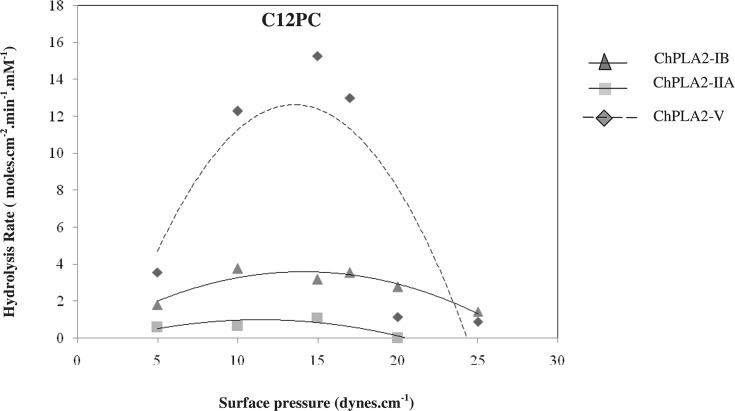
Variations in phospholipase activity of 1,2 DDPC monolayers with the surface pressure Chicken pancreatic sPLA2 (ChPLA2-IB represented by a triangle), intestinal sPLA2 (ChPLA2-IIA represented by a square) and pulmonary sPLA2 (ChPLA2-V represented by a diamond) (1 M) injected into the reaction compartment of a zero-order trough (volume, 130 ml; surface area, 108.5 cm^2^). Buffer: 10 mM Tris/HCl, pH 8, 150 mM NaCl, 21 mM CaCl_2_, and 1 mM EDTA. Activities are expressed as the number of moles of substrate hydrolysed per time unit (min) and surface unit (cm^2^) at the appropriate phospholipases’ concentrations. The activity values are presented as the means of triplicate experiments.

**Figure 2:**
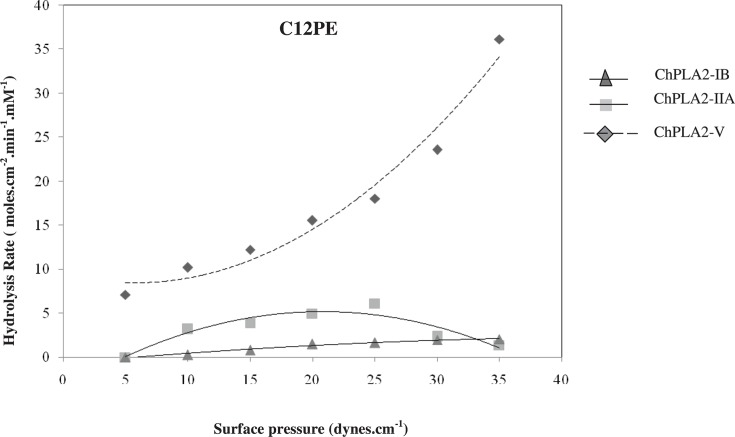
Variations in phospholipase activity of 1,2 DDPE, monolayers with the surface pressure ChPLA2-IB (represented by a triangle), ChPLA2-IIA (represented by a square) and pulmonary sPLA2 ChPLA2-V (represented by a diamond) (1 M) injected into the reaction compartment of a zero-order trough (volume, 130 ml; surface area, 108.5 cm^2^). Buffer: 10 mM Tris/HCl, pH 8, 150 mM NaCl, 21 mM CaCl_2_, and 1 mM EDTA. Activities are expressed as the number of moles of substrate hydrolysed per time unit (min) and surface unit (cm^2^) at the appropriate phospholipases concentrations. The activity values are presented as the means of triplicate experiments.

But when we compared the group V ChPLA2 activities with both pancreatic and intestinal sPLA2 from chicken, two major differences were observed. Firstly, the maximal activity of ChPLA2-V on 1,2 DDPC and 1,2 DDPE reached 15.26 and 36.12 moles/cm^2^.min.mM, respectively at a pressure of 15 and 35 dynes/cm, respectively. Whereas, those of ChPLA2-IB were 3.58 (at the pressure 20 dynes/cm) and 4.9 moles/cm^2^.min.mM (at the pressure 20 dynes/cm). This observation is confirmed by the activity of ChPLA2-IIA measured on PC and PE film showing that the activity was approximately 1.11 and 6.158 moles/cm^2^.min, Figures 1 and 2. Thus, ChPLA2-V seemed to be more active then the pancreatic and the intestinal chicken PLA2 on C12PC and C12PE.

**Figure 3:**
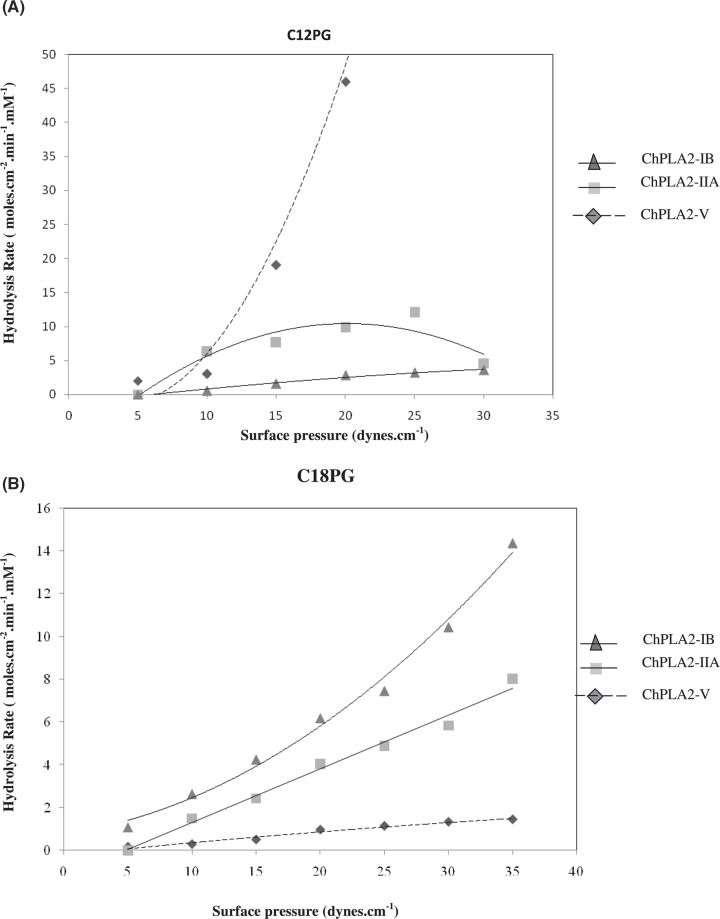
Variations in phospholipase activity of 1,2 DDPG and 1,2 POPG (**A**) Variations in phospholipase activity of 1,2 DDPG, monolayers with the surface pressure. ChPLA2-IB (represented by a triangle), ChPLA2-IIA (represented by a square) and ChPLA2-V (represented by a diamond) (1 M) injected into the reaction compartment of a zero-order trough (volume, 130 ml; surface area, 108.5 cm^2^). Buffer: 10 mM Tris/HCl, pH 8, 150 mM NaCl, 21 mM CaCl_2_, and 1 mM EDTA. Activities are expressed as the number of moles of substrate hydrolysed per time unit (min) and surface unit (cm^2^) at the appropriate phospholipases’ concentrations. The activity values are presented as the means of triplicate experiments. (**B**) Variations in phospholipase activity of 1,2 POPG, monolayers with the surface pressure. ChPLA2-IB (represented by a triangle), ChPLA2-IIA (represented by a square) and ChPLA2-V (represented by a diamond) (1 M) injected into the reaction compartment of a zero-order trough (volume, 130 ml; surface area, 108.5 cm^2^). Buffer: 10 mM Tris/HCl, pH 8, 150 mM NaCl, 21 mM CaCl_2_, and 1 mM EDTA. Activities are expressed as the number of moles of substrate hydrolysed per time unit (min) and surface unit (cm^2^) at the appropriate phospholipases concentrations. The activity values are presented as the means of triplicate experiments.

Second, we compared Phosphatidylglycerol (PG) with different fatty acid chains at the sn-2 position to examine the substrate preference for the ChPLA2-V. ChPLA2-V activity toward long- and short-fatty acid chains, at the sn-2 position was 1.45 moles/cm^2^.min.mM using 1- palmitoyl-2-oleoyl (POPG), and 122 moles/cm^2^.min when using 1,2 didodecanoyl phosphatidyl-glycerol (DDPG) (Figure 3A,B). When 1,2 POPG was used as substrate, 0.5 mM of β-CD, a lipolytic products acceptor, was added to the aqueous subphase. In fact, β-CD was used to solubilise water-insoluble long-chain lipolytic products (C18:1). In this work, we reported the variation of surface pressure of POPG with time, in the presence and in the absence of β-CD into the subphase, after stabilisation of the POPG on monolayer. During the kinetic measurements, all the activities tested in presence or in absence of β-CD, approved that surface pressure of POPG monolayers remains rather constant with time. Even when we injected β-CD into the subphase, the same behaviour of POPG was obtained, at all the pressures tested. These observations confirmed well that β-CD did not interact with this substrate. Whereas, ChPLA2IB and IIA showed a much higher enzymatic activity on long-chain phosphatidyl glycerol (POPG) which reached values of 14.3 and 10.5 moles/cm^2^.min.mM, respectively (Figure 3B). It is surprising to note that oleoyl-PG was not a good substrate for ChPLA2-V, although the cellular studies have shown that GV-PLA2 is involved in arachidonic acid release during the inflammatory process.

In trying to establish a structure–function relationship, we examined the surface electrostatic potentials of the various sPLA2 from chicken and mammals tested. Based on the total number of Lys, Arg, His, Asp and Glu, ChPLA2-IB has a net tabulated charge of +1 (+19, −18) whereas that of the ChPLA2-IIA calculated was +16 (+23, −7) and that of ChPLA2-V was +14 (+22, −8). Surface electrostatic potentials values may partially explain the relative preference of charged phospholipids (Supplementary Figure S1).

## Discussion

In this work, we reported, and for the first time the variations of surface pressure (O n) with time of three different head groups of phospholipids by chicken group V, IIA and IB PLA2 using the monomolecular film technique. The comparative study included both adsorption kinetics of pancreatic, intestinal and pulmonary sPLA2 and phospholipids hydrolysis.

All measurements were performed under the same hydrolytic conditions. Once the amounts of the appropriate enzyme are injected into the aqueous reaction subphase, the obtained curves relative to kinetic measurement demonstrated that the variation of surface pressure with time was principally the result of the phospholipid hydrolysis by sPLA2 at the interface [5,16].

Based on the electrostatic interactions, we can consider that the potential electrostatic is a crucial parameter for the interfacial binding of sPLA2. Thus, it was proposed that short-chain phosphatidylglycerol (C12PG), a negatively charged phospholipid, was the preferred substrate for most of the sPLA2 and this also explain the high catalytic activity of ChPLA2-IIA (+16) and ChPLA2-V (+14) on phosphatidylethanolamine (C12PE), a partial negatively charged substrate. Thus, and as a general conclusion, we can consider that ChPLA2-V was much more effective than the ChPLA2-IB and IIA tested, since it possessed the maximal specific activity among all the substrates tested. It was well established that tryptophan residue plays an important role in the binding capacity. In fact, on its interfacial binding surface, ChPLA2-IIA, which binds poorly to phosphatidylcholine, is devoid of tryptophan residues. The same behaviour was obtained with mammalian sPLA2 using phosphatidylcholine-rich vesicles [17,18]. At the same time, it has been reported that the addition of tryptophan to the membrane binding surface of hPLA2-IIA allows this enzyme to be more active on phosphatidylcholine-rich membranes [18].

As expected, ChPLA2-V demonstrated a high hydrolysis rate on phosphatigylglycerol as substrate, indicating its strong preference, as well as all sPLA2, for the negatively charged phospholipids. The same observation was concluded in a previous work when using pure phospholipid vesicles with human and mouse orthologues [17]. Interestingly, we noticed a high and continuous increase in activity of ChPLA2-V at an anionic interface, even at the highest surface pressure tested.

To examine the substrate preference of the group V-PLA2, deferent head groups and fatty acid chains on the phospholipid backbone were compared. Our results show the substrate preference of group V-PLA2 toward PG > PE > PC with the same fatty acid chains on the backbone (C12). These results are in a general agreement with the reported head group preference for this enzyme.

PGs with deferent fatty acid chains at the sn-2 position were compared to examine the substrate preference for the ChPLA2-V. For deferent fatty acid chains, activity towards short and long fatty acid chains at the sn-2 position was determined with C12PG and C18:1 PG (Figure 3A,B). In the presence of C12PG, the hydrolysis rate increased nearly 100-fold, but in the presence of C18:1 PG, a very weak activity was recorded, even with the the use of 0,5 mM β-CD. Thus, we can conclude that the fatty acid preference of ChPLA2-V is 2-decanoyl > 2-oleoyl with a PG head group and so ChPLA2-V much prefers short- than long-chain fatty acids. These results agree well with experiments with human group V sPLA2 (hPLA2-V). In fact, Chen and Dennis [19] demonstrate that the fatty acid preference of hPLA2-V is linoleoyls > palmitoyls > arachidonyl with a PC head group and sonicated vesicles. These results could be explained with the fact of the activity depends on the physical state of the substrate and natural membranes that contain a mixture of phospholipids may be more optimum. Several studies in mammals showed that distinct secreted PLA2 appear in lung cells and some are able to trigger molecular events leading to enhanced inflammation and lung damage causing the acute respiratory distress syndrome (ARDS) [20]. The latter is characterised by an alteration of pulmonary surfactant which increases surface tension at the air liquid interface. sPLA2-IIA, -V, and X can directly hydrolyse lung surfactant phospholipids [20]. It was recently demonstrated that only hPLA2-V and -X hydrolyse the pulmonary surfactant [21]. Indeed, hPLA2-V was considered as the principal sPLA2 implicated in ARDS. When transgenic mice over expressing group V sPLA2 were used, the immediate death after birth was obtained which was not observed in mice overexpressing mPLA2-X. This is due to the alteration of the surfactant composition [22].

Supplementary structural studies and mutagenesis on some key residues might better explain our data, especially on the clear preference of chicken sPLA2 group V to hydrolyse mainly negative head groups phospholipids (PG and PE) and on its capacity to act on short-chain fatty sacid on the phospholipid backbone, in order to gain more structural–function relationship of ChPLA2-V.

## Supplementary Material

Supplementary Figure S1

## Abbreviations

ChPLA2-IB: pancreatic chicken sPLA2
ChPLA2-IIA: chicken intestinal sPLA2
ChPLA2-V: pulmonary V sPLA2
hPLA2-V: human group V sPLA2
Pc: phosphatidyl-cholin
PE: phosphatidyl-ethonolamin
sPLA2: secreted phospholipase A2
1,2 DDPC: l-2 didodecanoyl-sn-glycero-3-phosphatidylcholine
1,2 DDPE: 1,2 didodecanoyl-sn-3-phosphatidy-ethanolamine
1,2 POPG: 1 palmitoyl-2-oleoyl-sn-3-phosphatidyl-glycerol
β-CD: β-cyclodextrin

## References

[1] Schaloske R.H. and Dennis E.A. (2006) The phospholipase A2 superfamily and its group numbering system. Biochim. Biophys. Acta 1761, 1246–1259 10.1016/j.bbalip.2006.07.011 16973413

[2] Lambeau G. and Gelb M.H. (2008) Biochemistry and physiology of mammalian secreted phospholipases A2. Annu. Rev. Biochem. 77, 495–520 10.1146/annurev.biochem.76.062405.154007 18405237

[3] Valentin E. et al. (2000) Novel human secreted phospholipase A(2) with homology to the group III bee venom enzyme. J. Biol. Chem. 275, 7492–7496 10.1074/jbc.275.11.7492 10713052

[4] Rouault M., Bollinger. J.G. et al. (2003) Novel mammalian group XII secreted phospholipase A2 lacking enzymatic activity. Biochemistry 42, 11494–11503 10.1021/bi0349930 14516201

[5] Singer A. et al. (2002) Interfacial kinetic and binding properties of the complete set of human and mouse groups I, II, V, X, and XII secreted phospholipases A2. J. Biol. Chem. 277, 48535–48549 10.1074/jbc.M205855200 12359733

[6] Valentin E. et al. (1999) On the diversity of secreted phospholipases A(2). Cloning, tissue distribution, and functional expression of two novel mouse group II enzymes. J. Biol. Chem. 274, 31195–31202 10.1074/jbc.274.44.31195 10531313

[7] Scott K.F., Graham G.G. and Bryant K.J. (2003) Secreted phospholipase A2 enzymes as therapeutic targets. Expert Opin. Ther. Targets 7, 427–440 10.1517/14728222.7.3.427 12783578

[8] Hanasaki K., Ono T. et al. (1999) Purified group X secretory phospholipase A(2) induced prominent release of arachidonic acid from human myeloid leukemia cells. J. Biol. Chem. 274, 34203–34211 10.1074/jbc.274.48.34203 10567392

[9] Chen J. et al. (1994) Cloning and recombinant expression of a novel human low molecular weight Ca^2+^-dependent phospholipase-A(2). J. Biol. Chem. 269, 2365–2368 8300559

[10] Chen J. et al. (1994) Cloning and characterization of novel rat and mouse low molecular weight Ca2+-dependent phospholipase A(2)s containing 16 cysteines. J. Biol. Chem. 269, 23018–23024 8083202

[11] Dennis E.A. (1997) The growing phospholipase A2 superfamily of signal transduction enzymes. Trends Biochem. Sci. 22, 1–2 10.1016/S0968-0004(96)20031-3 9020581

[12] Karray A. et al. (2009) Biochemical and molecular characterization of purified chicken pancreatic phospholipase A2. FEBS J. 276, 4545–4554 10.1111/j.1742-4658.2009.07160.x 19645724

[13] Karray A. et al. (2011) Purification and biochemical characterization of a secreted group IIA chicken intestinal phospholipase A2. Lipids Health Dis. 10, 27 10.1186/1476-511X-10-27 21284884 PMC3040156

[14] Brockman H.L. (1984) General features of lipolysis: reaction scheme, interfacial structure and experimental approaches. In Lipases(Borgström B. and Brockman H.L., eds), pp. 3–46, Elsevier, Amsterdam

[15] Pattus F., Slotboom A.J. and de Haas G.H. (1979) Regulation of Phospholipase A2 Activity by the Lipid -Water Interface: a Monolayer Approach. Biochemistry 13, 2691–2697 10.1021/bi00580a001 573135

[16] Verger R. and de Haas G.H. (1973) Enzyme reactions in a membrane model. 1: a new technique to study enzyme reactions in monolayers. Chem. Phys. Lipids 10, 127–136 10.1016/0009-3084(73)90009-1 4698875

[17] Bezzine S. et al. (2002) On the binding preference of human groups IIA and X phospholipases A2 for membranes with anionic phospholipids. J. Biol. Chem. 277, 48523–48534 10.1074/jbc.M203137200 12244093

[18] Bezzine S. et al. (2000) Exogenously added human group X secreted phospholipase A(2) but not the group IB, IIA, and V enzymes efficiently release arachidonic acid from adherent mammalian cells. J. Biol. Chem. 275, 3179–3191 10.1074/jbc.275.5.3179 10652303

[19] Chen Y. and Dennis E. (1998) Expression and characterization of human group V phospholipase A2. Biochim. Biophys. Acta 1394, 57–64 10.1016/S0005-2760(98)00098-8 9767110

[20] Kitsiouli E., Nakos G. and Lekka M.E. (2009) Phospholipase A2 subclasses in acute respiratory distress syndrome. Biochim. Biophys. Acta 1792, 941–953 10.1016/j.bbadis.2009.06.007 19577642

[21] Chabot S. et al. (2003) Inhibitory effects of surfactant protein A on surfactant phospholipid hydrolysis by secreted phospholipases A2. J. Immunol. 171, 995–1000 10.4049/jimmunol.171.2.995 12847272

[22] Ohtsuki M. et al. (2006) Transgenic expression of group V, but not group X, secreted phospholipase A2 in mice leads to neonatal lethality because of lung dysfunction. J. Biol. Chem. 281, 36420–36433 10.1074/jbc.M607975200 17008322

[23] Kurray A., Zarai Z., Gargouri Y., Verger R., and Bezzine S. (2011) kinetic properties of pancreatic and intestinal sPLA2 from chicken and mammls using the monomolecular film technique. J. Colloid Interface Sci. 363, 620–625 21855891 10.1016/j.jcis.2011.07.041

