# Supplementary material for: Hydrolysis of three different head groups phospholipids by chicken group V phospholipase A2 using the monomolecular film technique
Source: Biosci Rep. 2020 Jan 21;40(1):BSR20192053. doi: 10.1042/BSR20192053 (PMC6974423; doi:10.1042/BSR20192053)
Supplement: Supplementary Figure S1 [file BSR-2019-2053_supp.pdf]

Supplementary file.

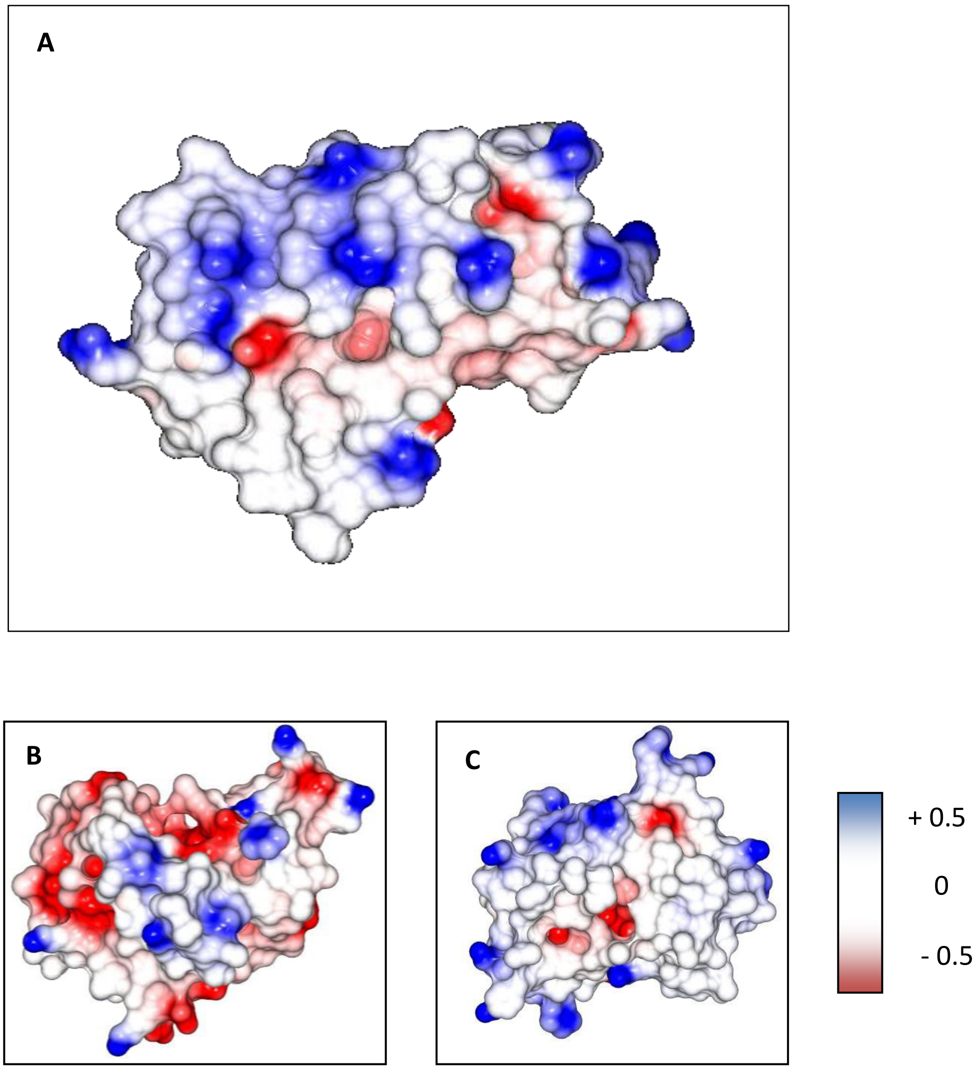

Supplementary figure S1 Surface GRASP representations of ChPLA2-V (A), ChPLA2-IB (B) and ChPLA2-IIA (C). Using the CCP4MG programme, the potential substrate binding surfaces are represented by Van der Waals's colored code where red and blue represent a negative net charge and positive charge, respectively. Whereas, white colour represent the total neutral positions.
